# Accelerated DNA vaccine regimen provides protection against Crimean-Congo hemorrhagic fever virus challenge in a macaque model

**DOI:** 10.1016/j.ymthe.2022.09.016

**Published:** 2022-10-03

**Authors:** David W. Hawman, Kimberly Meade-White, Shanna Leventhal, Sofia Appelberg, Gustaf Ahlén, Negin Nikouyan, Chad Clancy, Brian Smith, Patrick Hanley, Jamie Lovaglio, Ali Mirazimi, Matti Sällberg, Heinz Feldmann

**Affiliations:** 1 Laboratory of Virology, Division of Intramural Research, National Institute of Allergy and Infectious Diseases, National Institutes of Health, Rocky Mountain Laboratories, Hamilton, MT 59840, USA; 2 Rocky Mountain Veterinary Branch, Division of Intramural Research, National Institute of Allergy and Infectious Diseases, National Institutes of Health, Rocky Mountain Laboratories, Hamilton, MT 59840, USA; 3 Division of Clinical Microbiology, Department of Laboratory Medicine, Karolinska Institutet, 141 86 Stockholm, Sweden; 4 Public Health Agency of Sweden, 171 82, Solna, Sweden; 5 National Veterinary Institute, 751 89 Uppsala, Sweden

**Keywords:** Crimean-Congo hemorrhagic fever, CCHF, vaccine, DNA vaccine, macaque model, correlates of protection

## Abstract

Crimean-Congo hemorrhagic fever virus (CCHFV) is widely distributed throughout Africa, the Middle East, Southern Asia, and Southern and Eastern Europe. Spread by *Hyalomma* ticks or by contact with infected animals, CCHF begins non-specifically but can rapidly progress to severe, sometimes fatal, disease. Due to the non-specific early symptoms and often unrecognized infections, patients often present to healthcare systems exhibiting later stages of disease, when treatment is limited to supportive care. Consequently, simple vaccines are critically needed to protect populations at risk of CCHFV infection. Currently, there are no widely approved vaccines for CCHFV. We have previously reported significant efficacy of a three-dose DNA-based vaccination regimen for CCHFV in cynomolgus macaques (*Macaca fasicularis)*. Here, we show that in cynomolgus macaques, plasmid-expressed CCHFV nucleoprotein (NP) and glycoprotein precursor (GPC) antigens elicit primarily humoral and cellular immunity, respectively. We found that a two-dose vaccination regimen with plasmids expressing the NP and GPC provides significant protection against CCHFV infection. Studies investigating vaccinations with either antigen alone showed that plasmid-expressed NPs could also confer protection. Cumulatively, our data show that this vaccine confers robust protection against CCHFV and suggest that both humoral and cellular immunity contribute to optimal vaccine-mediated protection.

## Graphical abstract

**Figure:**
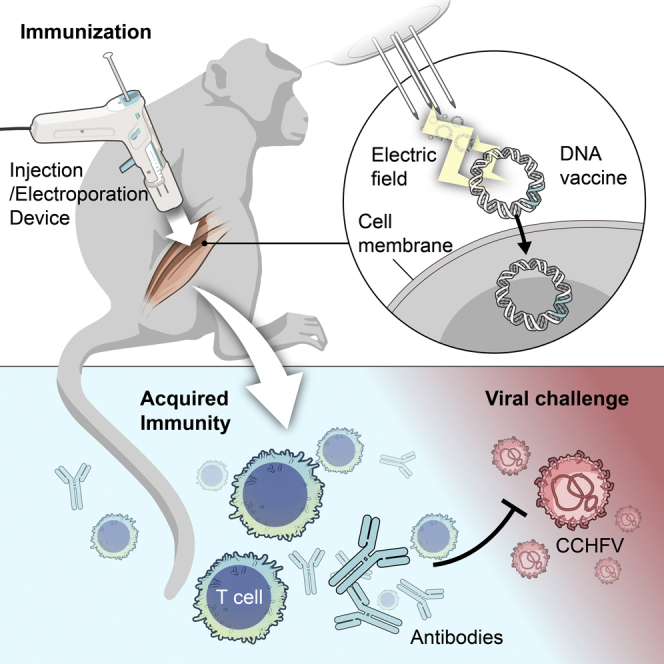

## Teaser

Feldmann and colleagues have evaluated a DNA-based vaccine for Crimean-Congo hemorrhagic fever virus in a non-human primate model and found that two immunizations were sufficient to protect against infection and disease. Protection correlated with a robust, but non-neutralizing, antibody response. These data support continued development of this vaccine candidate.

## Introduction

Crimean-Congo hemorrhagic fever (CCHF) is a widely distributed tick-borne febrile illness. The geographic range of the CCHF virus (CCHFV) closely follows the range of the *Hyalomma* tick vector, and cases are reported throughout Africa, Southern and Eastern Europe, the Middle East, and Asia.^1^^,^^2^ CCHF typically begins as a non-specific febrile illness but can progress to severe, hemorrhagic manifestations.^3^ Common predictors of poor outcome are high viral loads, elevated liver enzymes, thrombocytopenia, elevated inflammatory cytokines, and absent antibody responses.^3^^,^^4^^,^^5^ Despite cases of CCHFV being reported annually, there are no widely approved vaccines or approved therapeutics for CCHF. The World Health Organization lists CCHFV as a priority pathogen for countermeasure development.^6^ Ribavirin has been used with unclear efficacy,^7^ and a Bulgarian vaccine appears immunogenic in humans.^8^ However, this vaccine is prepared from inactivated virus grown in mouse brain homogenates, making wide approval unlikely. Several vaccines for CCHFV have been evaluated in rodent models^9^^,^^10^^,^^11^ with varying efficacies from complete to no protection. These vaccines have largely focused on the CCHFV glycoproteins (Gn and Gc) glycoprotein precursor (GPC) and/or nucleoprotein (NP) as vaccine-encoded antigens. However, the correlates of protection for CCHFV vaccines remain unclear. Notably, neutralizing antibodies appear dispensable for vaccine-mediated protection.^10^^,^^12^^,^^13^

We recently reported on the first vaccine to demonstrate efficacy against CCHFV in the cynomolgus macaque model.^12^^,^^14^ We showed that *in vivo* electroporation of DNA plasmids encoding the NP (pNP) and GPC (pGPC) in a prime-boost-boost regimen conferred significant protection against viral replication and disease in macaques infected with CCHFV.^12^ We report here an advanced understanding of the protective mechanism of this vaccine and an important refinement for public health use. We show that two immunizations of this vaccine platform with both the NP and GPC antigens confer significant protection against CCHFV in infected macaques. Interestingly, we found that this combined vaccination with both pNP and pGPC conferred superior protection against CCHFV infection than even three immunizations with either pNP or pGPC alone. Our results suggest that immunity to both antigens and both humoral and cellular immunity may contribute to vaccine-mediated protection against CCHFV.

## Results

### Study design

**Figure 1:**
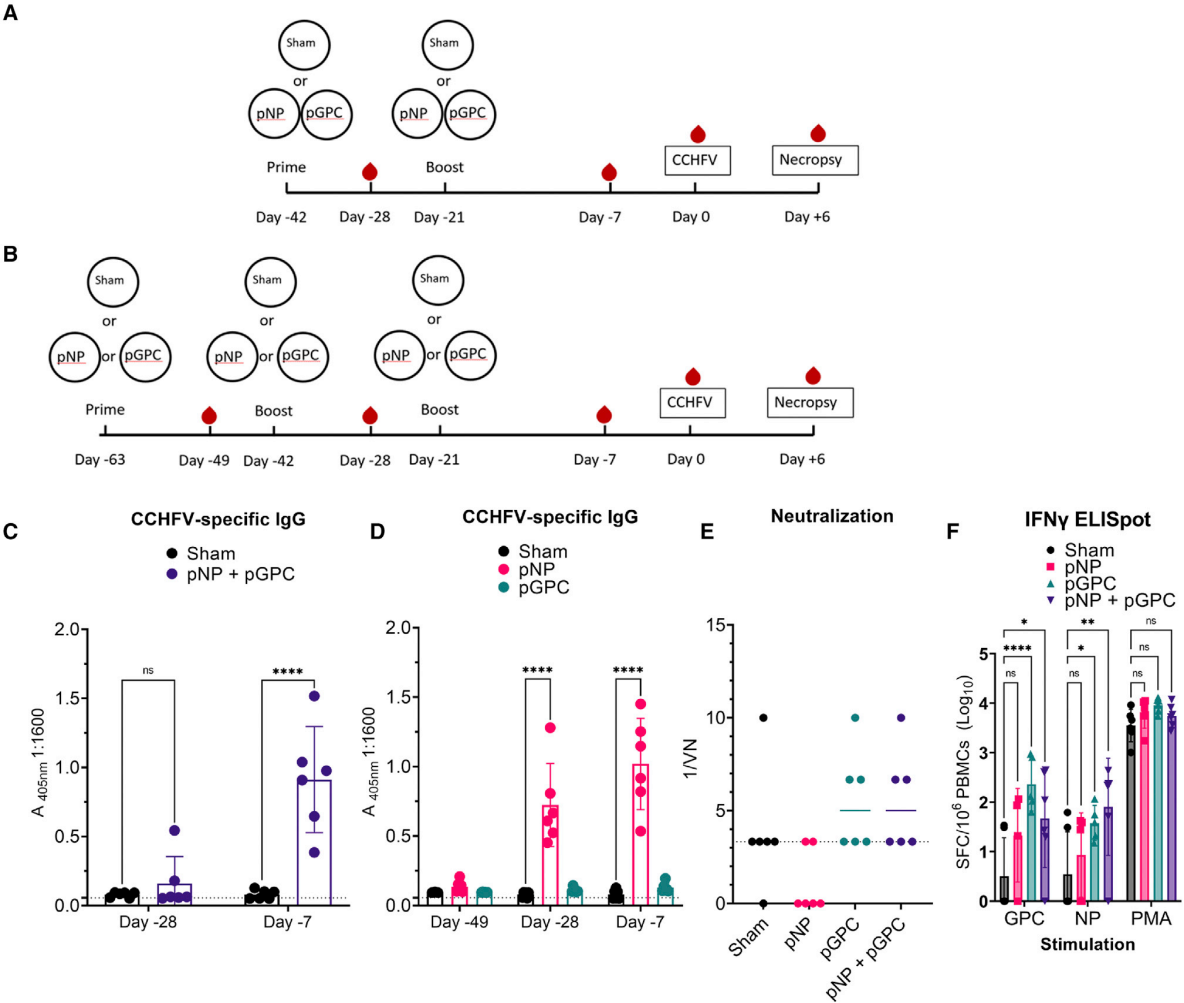
Study design and immunogenicity of pNP and pGPC vaccinations In the first study, 3 macaques were vaccinated with an empty plasmid (sham), or 6 macaques were vaccinated with both pNP and pGPC plasmids (pNP + pGPC). Three weeks later, animals were boosted with identical vaccinations and challenged with CCHFV 3 weeks after boosting (A). In the second study, 3 macaques were sham vaccinated as before or received vaccinations of pNP or pGPC alone. This study was performed as a prime-boost-boost at 3 week intervals followed by CCHFV challenge 3 weeks after last vaccination (B). For both studies, blood draws were performed to collect serum 2 weeks after each vaccination and PBMCs collected on day −7 to monitor immune responses to the vaccine. CCHFV-specific IgG in serum was quantified by whole-virion ELISA (C and D). Serum neutralization activity measured at day −7 via a microneutralization assay against infectious CCHFV (E). The y axis represents the maximum dilution at which no infectious virus was observed after incubation with serum. CCHFV-specific T cell responses were measured by ELISpot (F). Data presented as cumulative spot-forming cells (SFCs) measured against overlapping peptide pools spanning the entire GPC or NP after subtracting SFCs measured in DMSO-only stimulated wells. Counts were normalized to 10^6^ PBMCs. As positive control, cells were stimulated with PMA/ionomycin (PMA). p values were calculated with two-way ANOVA with Dunnett’s multiple comparison test (C, D and F) or One-way ANOVA with Dunnet’s multiple comparison test (E). NS p > 0.05, ∗p < 0.05, ∗∗p < 0.01, ∗∗∗∗p < 0.0001. Error bars indicate mean ± SD.

The data reported herein were from two sequential studies comprising 24 total cynomolgus macaques. Animals were randomly assigned to study groups, and the date of birth, starting weight, and sex of animals in each group are provided in Table S1. In the first study, a group of 6 cynomolgus macaques was vaccinated intramuscularly with 1 mg each of a plasmid expressing the CCHFV NP and a plasmid expressing the CCHFV GPC (pNP + pGPC, cumulative dose of 2 mg DNA), and 3 cynomolgus macaques received 1 mg empty plasmid (sham). Three weeks later, animals were boosted with an identical vaccination, and 3 weeks after boosting, animals were challenged with CCHFV (Figure 1A). Vaccination was delivered via an intramuscular injection followed by *in vivo* electroporation as previously described.^12^ Based on immunogenicity and efficacy data obtained with both antigens in a prime-boost approach, in the second study, we elected to perform a prime-boost-boost strategy for evaluating the single antigen approach. Groups of 6 cynomolgus macaques were vaccinated with 1 mg of either the pNP or the pGPC plasmid, and a group of 3 cynomolgus macaques were sham vaccinated. Three and six weeks later, animals were boosted with identical vaccinations. Three weeks after the last boost, animals were challenged with CCHFV (Figure 1B). Both studies were pooled for data presentation and analyses. Vaccination appeared well tolerated with no serious adverse events following vaccination.

### Vaccination with pNP, but not pGPC, results in CCHFV-specific IgG response

To evaluate immunogenicity of these vaccination approaches, sera were evaluated for CCHFV-specific immunoglobulin G (IgG) via whole-virion ELISA. Two weeks after prime vaccination, neither the combination of pNP and pGPC nor the individual plasmids resulted in a significant CCHFV-specific IgG response (Figures 1C and 1D). After boosting, only animals receiving the pNP vaccination with or without pGPC had a detectable CCHFV-specific IgG response (Figures 1C and 1D). Animals receiving just the pGPC vaccination had an ELISA signal similar to sham-vaccinated animals (Figure 1D) even after three immunizations, suggesting that the pGPC vaccination failed to elicit a CCHFV-specific IgG response. Interestingly, the pNP and pNP + pGPC groups had nearly identical CCHFV-specific IgG responses 2 weeks after their third and second immunizations, respectively (Figures 1C and 1D), suggesting that the prime-boost-boost approach did not improve CCHFV-specific antibody responses over just a prime-boost approach. These data are consistent with our previous report showing similar antibody titers post-prime-boost versus post-prime-boost-boost.^12^ Lastly, consistent with our data showing that pNP, but not pGPC, elicited a humoral response, compared with sham-vaccinated animals, we observed no significant serum neutralizing activity against infectious CCHFV from sera collected at time of CCHFV challenge in any group (Figure 1E). These data are similar to our previous data showing little to no neutralizing activity in vaccinated animals^12^ and support the hypothesis that humoral immunity is primarily directed toward NP.

We also evaluated T cell responses to vaccination via an interferon γ (IFNγ) ELISpot on peripheral blood mononuclear cells (PBMCs) collected 2 weeks after the last vaccination for each group (Figure 1F). Compared with sham-vaccinated animals, increases in spot-forming cells (SFCs) were seen in pNP-only-vaccinated animals against either the GPC or NP peptide pools; however, this was not statistically significant (p > 0.05) (Figure 1G). Compared with sham-vaccinated animals, statistically significant increases in SFCs were measured in pGPC animals against the GPC peptides, and pNP + pGPC animals had significant responses against both the GPC and NP peptide pools (Figure 1G). Nevertheless, our ELISpot data indicated that cellular immunity in response to pNP and/or pGPC vaccination was low. Cumulatively, our data showed that pNP or pNP + pGPC vaccination induced a robust, but non-neutralizing, CCHFV-specific IgG response with relatively weaker cellular immunity.

### A two-dose pNP + pGPC vaccine regimen provides protection against clinical disease

**Figure 2:**
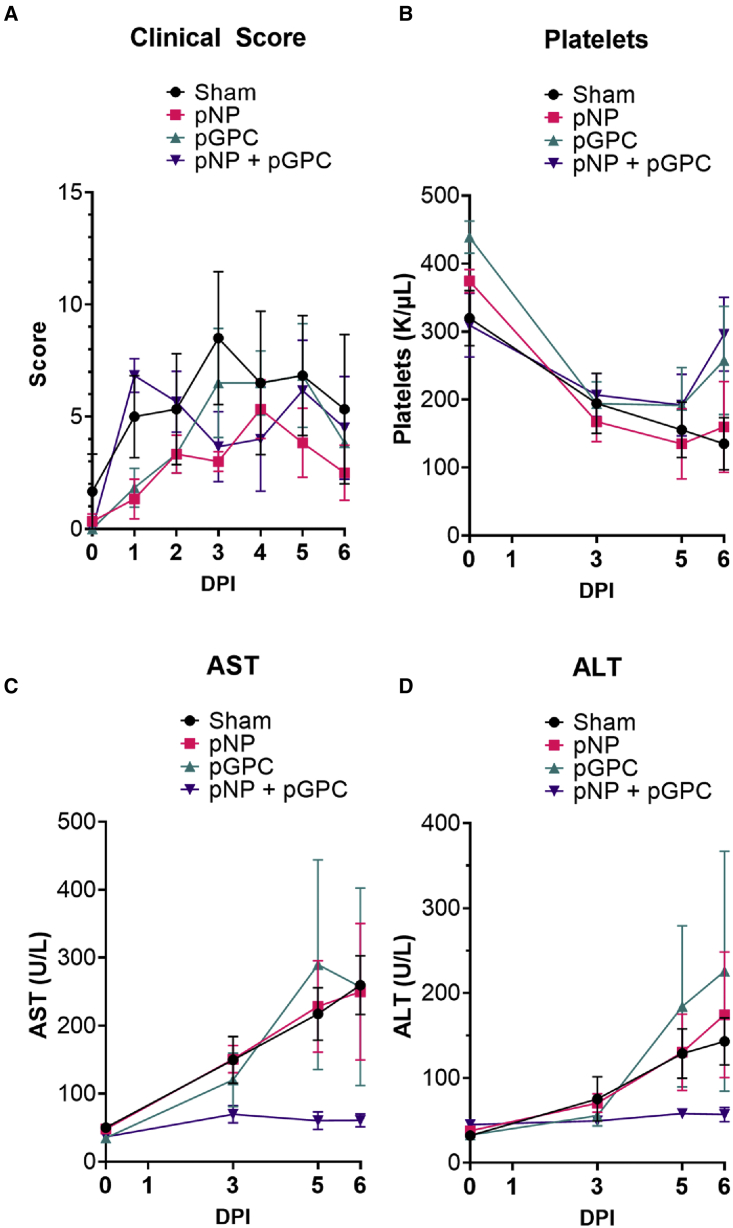
pNP + pGPC vaccination protects against clinical disease following CCHFV infection Vaccinated macaques were challenged with CCHFV via a combined subcutaneous (s.c.)/intraperitoneal (i.p.) route. Animals were comprehensively scored daily (A), and on days 0, 1,3, 5, and 6, blood draws were conducted to measure blood chemistry and hematology (B–D). AST, aspartate aminotransferase; ALT alanine aminotransferase. Error bars indicate ± SEM. The complete blood chemistry and hematology is provided in the supplemental information.

To evaluate efficacy of the vaccinations against CCHFV we challenged the animals with 1 × 10^5^ TCID_50_ of CCHFV strain Hoti via a combined subcutaneous and intravenous inoculation.^14^ Animals were scored for clinical disease daily, and on days 0, 3, 5, and 6 post-infection (PI), we collected blood and sera for evaluation of blood chemistry and hematology. Overall, clinical disease in animals of any group was low (Figure 2A), although it was usually highest in the sham-vaccinated group. No significant differences (p > 0.05) in scores were seen between groups (Figure 2A). The most common clinical sign was decreased appetite and mild lethargy. No animals reached euthanasia criteria during the course of the study. Thrombocytopenia is commonly reported in human CCHF cases^3^ and non-human primates (NHPs) infected with CCHFV.^12^^,^^14^ Compared with their respective starting values on day 0, the sham and pNP groups both had significantly reduced platelet counts on days 5 and 6, while the pGPC group had significantly reduced platelet counts by day 3 onward (Figure 2B; two-way ANOVA with Dunnett’s multiple comparison test). In contrast, the pNP + pGPC group had no significant (p > 0.05) changes in platelet counts (Figure 2B). Elevated liver enzymes such as AST and ALT are also a commonly reported correlate of poor outcome.^3^^,^^4^ Compared with their respective starting values on day 0, the pGPC group had significantly (p < 0.05) increased AST on days 3 and 5 and increased ALT on day 5 (Figures 2C and 2D; two-way ANOVA with Dunnett’s multiple comparison test), while AST and ALT trended higher in the sham and pNP groups following infection (Figures 2C and 2D). However, the increases in AST and ALT values in the pNP- and sham-vaccinated groups were not significant compared with their respective starting values on day 0 (Figures 2C and 2D; two-way ANOVA with Dunnett’s multiple comparison test). In contrast, AST and ALT values in pNP + pGPC-vaccinated animals showed no increases over the course of the study (Figures 2C and 2D). Cumulatively, these data indicate that a two-dose vaccine regimen with the combined pNP + pGPC vaccination protects against clinical disease following CCHFV infection, while protection afforded by pNP or pGPC alone is incomplete.

### The two-dose pNp + pGPC vaccination regimen provides rapid control of CCHFV viremia

**Figure 3:**
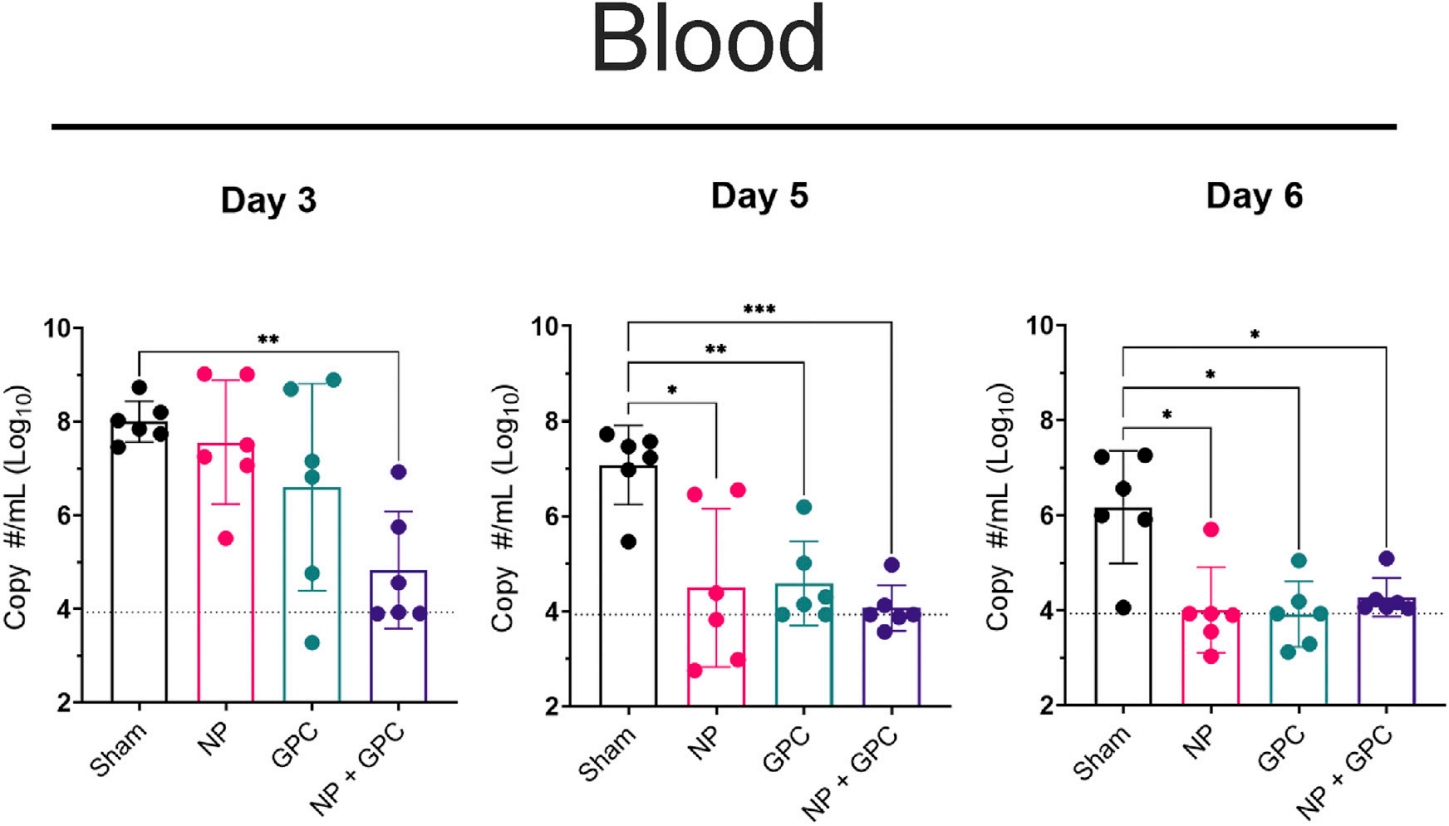
pNP + pGPC vaccination provides earlier control of viremia Viral RNA was measured in the blood on days 3, 5, and 6 PI via qRT-PCR. p values were calculated with Brown-Forsythe and Welch ANOVA with Dunnett’s multiple comparison test. Non-significant comparisons (p > 0.05) are not shown. ∗p < 0.05, ∗∗p < 0.01, ∗∗∗p < 0.001, ∗∗∗∗p < 0.0001. Dashed line indicates limit of quantitation. Error bars indicate mean ± SD.

To evaluate the kinetics of viral control in vaccinated and CCHFV-challenged animals, we collected blood and oral and nasal swabs on days 3, 5, and 6 PI. On day 3 PI, only pNP + pGPC-vaccinated animals had significantly reduced viremia compared with sham-vaccinated animals (Figure 3A). However, by days 5 and 6 PI, all groups of CCHFV-vaccinated animals had significantly reduced viremia compared with sham-vaccinated animals, indicating that these animals had slightly delayed control of CCHFV viremia (Figure 3A). In the oral and nasal swabs, viral burden remained low, even among sham-vaccinated animals, and no significant differences were seen among study groups (data not shown). This contrasts with our previous reports on CCHFV infection in NHPs in which viral RNA in nasal and oral swabs was readily detected.^12^^,^^14^

### Two-dose pNP + pGPC vaccination confers robust control of viral burdens in key tissues

**Figure 4:**
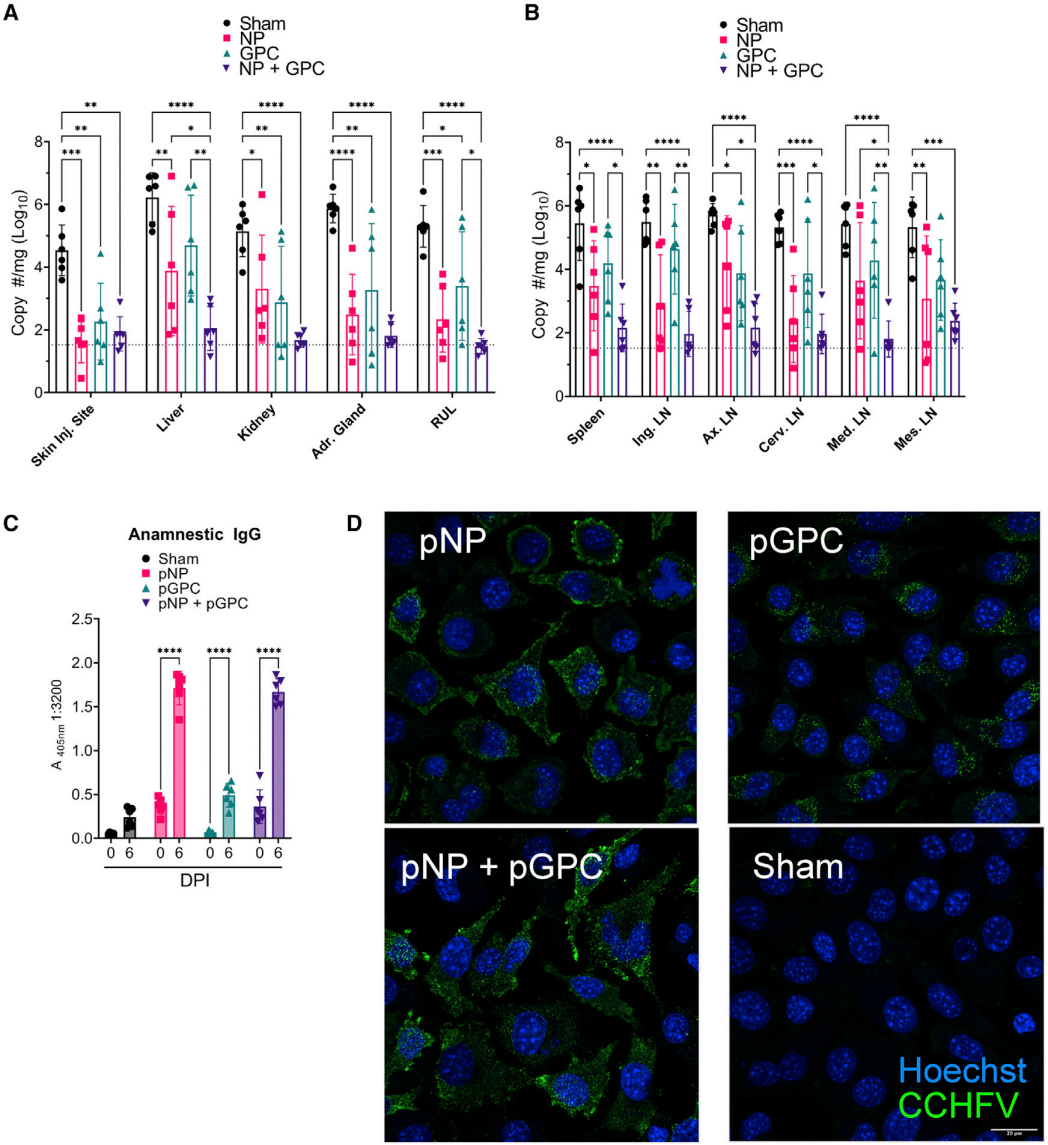
Vaccination significantly reduced CCHFV burden in multiple tissues Viral RNA in indicated tissues at day 6 PI was measured by qRT-PCR (A and B). RUL, right upper lung lobe. p values were calculated with two-way ANOVA with Tukey’s multiple comparisons test. Non-significant comparisons (p > 0.05) are not shown. Dashed line indicates limit of quantitation. Anamnestic CCHFV-specific IgG in serum collected at day 0 or 6 was measured by whole-virion ELISA (C). Serum collected at day 6 PI was evaluated for immunofluorescence against CCHFV-infected cells (D). Scale bar = 20 μm. p values were calculated with two-way ANOVA with Tukey’s multiple comparison test (A and B) or Sidak's multiple comparison test (C). ∗p < 0.05, ∗∗p < 0.01, ∗∗∗p < 0.001, ∗∗∗∗p < 0.0001.

To evaluate the efficacy of vaccination on controlling CCHFV infection, we performed a timed necropsy on day 6 PI and collected key target tissues of CCHFV replication. We found that compared with sham-vaccinated animals, in most non-lymphoid tissues, all three CCHFV-vaccinated groups had significantly reduced viral RNA burdens (Figure 4A). Notably, however, pGPC vaccination did not significantly reduce viral RNA in the liver, a key site of CCHFV-induced damage in humans. Importantly, both pNP- and pNP + pGPC-vaccinated groups, compared with sham-vaccinated animals, had significantly reduced viral RNA in the liver (Figure 4A). pNP + pGPC-vaccinated animals had significantly reduced viral RNA compared with pNP-vaccinated animals in the liver (Figure 4A), suggesting that pNP + pGPC vaccination conferred greater control of viral replication in this organ. This is despite pGPC vaccination alone having no significant effect. In lymphoid tissue, compared with sham-vaccinated animals, only pNP- or pNP + pGPC-vaccinated animals had significantly reduced viral RNA (Figure 4B). Viral loads in the spleen and lymph nodes were similar (p > 0.05) between sham- and pGPC-vaccinated animals (Figure 4B), suggesting that pGPC-only vaccination was insufficient to confer control of viral replication in lymphoid tissue. Similar to the liver, in the axillary and mediastinal lymph node, pNP + pGPC-vaccinated animals had significantly reduced viral RNA compared with pNP-only-vaccinated animals, suggesting that pNP + pGPC vaccination conferred greater control of the virus in these tissues. Across nearly all tissues evaluated, viral loads in pNP + pGPC-vaccinated animals trended lower than viral loads in pNP-only-vaccinated animals (Figures 4A and 4B), cumulatively suggesting that immune responses to both pNP and pGPC antigens may confer superior control of the CCHFV infection.

To further evaluate how well the viral infection was controlled, we evaluated the anamnestic CCHFV-specific antibody responses on day 6 PI. Compared with titers at day 0 PI, in all CCHFV-vaccinated animals, CCHFV infection resulted in a significant anamnestic antibody response, suggesting sufficient viral replication occurred to boost the pre-existing immunity (Figure 4C). Notably, in the pGPC group, in which we found no detectable CCHFV-specific antibody following vaccination and prior to CCHFV infection (Figure 1), we observed significantly increased CCHFV-specific IgG at day 6 PI (Figure 4C). Furthermore, robust labeling of CCHFV-infected cells was seen with sera from all vaccinated groups, including pGPC, but not sham-vaccinated animals (Figure 4D), suggesting that pGPC vaccination primed the host to rapidly respond to CCHFV.

### pNP or pGPC vaccination protects against tissue damage in the liver and spleen

**Figure 5:**
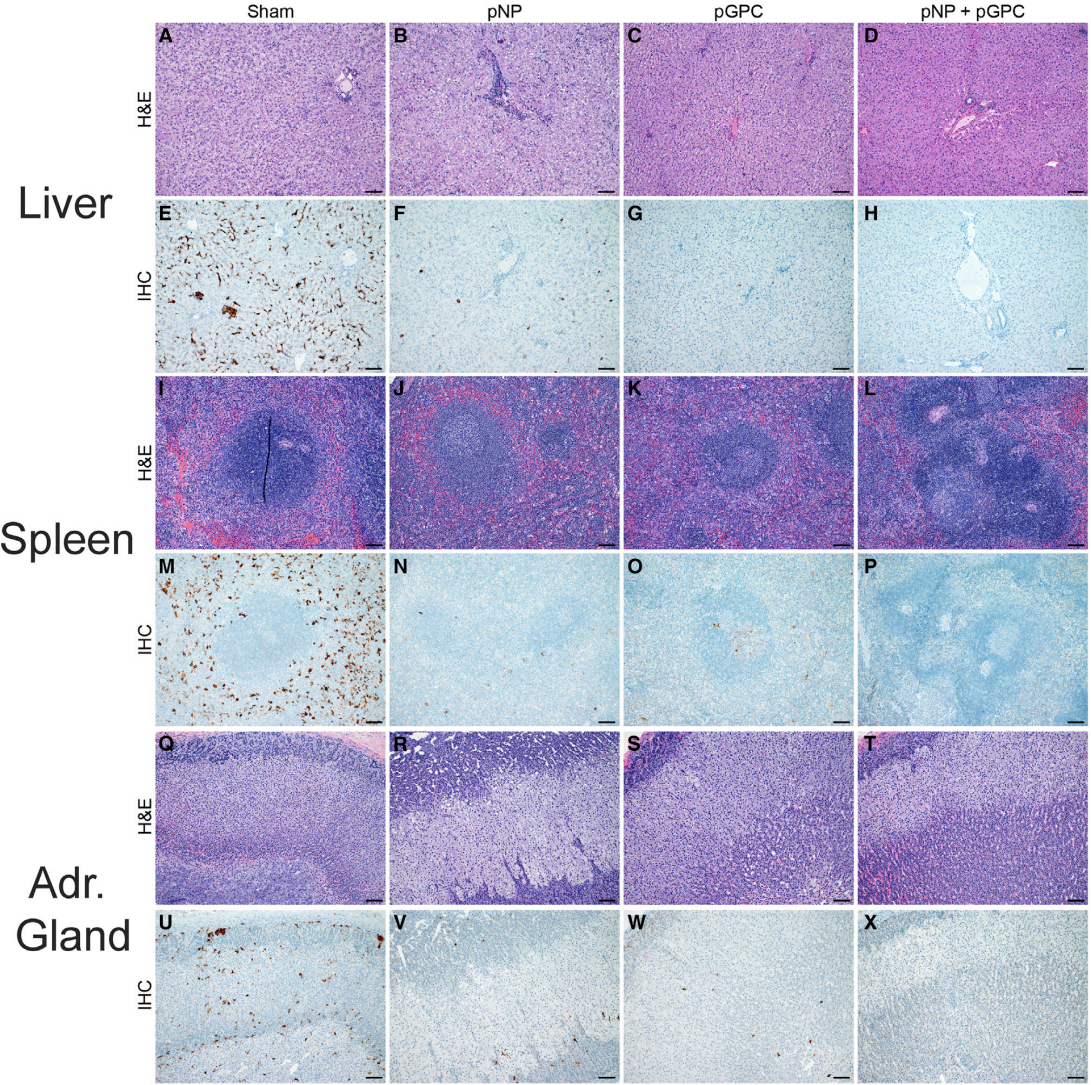
Histopathological findings in vaccinated animals At time of necropsy, tissues were formalin fixed, paraffin embedded and sectioned. Sections of the liver (A–H), spleen (I–P), and adrenal gland (Q–X) were stained with hematoxylin and eosin (H&E) (A–D, I–L, and Q–T) or for viral antigen (E–H, M–P, and U–X). Representative images from each group are shown. Rare, randomly scattered foci of mild hepatocellular necrosis (A). Rare foci of inflammation and individual hepatocellular necrosis (B). Rare, scattered aggregates of histiocytic cells and lymphocytes with minimal cellular debris (C). No observable change (D). Widely distributed CCHF immunoreactivity in Kupffer cells, endothelial cells, and moderate numbers of hepatocytes (E). Low numbers of CCHF immunoreactive Kupffer cells with fewer immunoreactive hepatocytes (F). Rare CCHF immunoreactivity in Kupffer cells (G). No observable CCHF immunoreactivity (H). Mild lymphoid depletion (I–K). Well-popular lymphoid follicles and peri-arteriolar lymphoid sheaths (L). Widespread CCHF immunoreactivity in histiocytic cells of both red pulp and lymphoid follicles (M). Rare CCHF immunoreactivity in histiocytic cells (N and O). No observable CCHF immunoreactivity (P). No significant histologic lesions observed in the adrenal gland (Q–T). Low to moderate numbers of endocrine cells show CCHF immunoreactivity in both the adrenal cortex and medulla (U–W). No observable immunoreactivity observed (X). Scale bar: 200 μm.

To evaluate if the reduction in viral loads seen in the liver and spleen were associated with reduced tissue damage, formalin-fixed sections of liver and spleen were evaluated for pathology and presence of viral antigen. Cumulatively, liver lesions were mild, with most animals, regardless of group, exhibiting no evidence of liver changes (Figure 5; Table 1). In the sham-vaccinated group, two animals had mild hepatocellular necrosis, while in the pNP or pGPC groups, one animal in each group had mild or moderate necrosis, respectively (Figure 5; Table 1). No liver lesions were seen in the group that received the pNP + pGPC vaccination (Figure 5; Table 1). In the spleen, 4 of 6 sham-vaccinated animals had lymphoid depletion, while no animals in the pNP, two in the pGPC, and one in the pNP + pGPC group had evidence of lymphoid depletion (Figure 5; Table 1). Two animals in the sham-vaccinated group also had evidence of interstitial pneumonia at time of necropsy (Table 1).

**Table 1:** Histological findings in CCHFV infected macaques

	Lesion prevalence			
	Sham, %	pNP	pGPC	pNP + pGPC
Lung interstitial pneumonia	33.3 (2/6)	0 (0/6)	0 (0/6)	0 (0/6)
Liver hepatocellular necrosis	33.3 (2/6)	33.3 (2/6)	16.7 (1/6)	0 (0/6)
Mild	33.3 (2/6)	16.7 (1/6)	0 (0/6)	0 (0/6)
Moderate	0 (0/6)	16.7 (1/6)	16.7 (1/6)	0 (0/6)
Spleen lymphoid depletion	66.6 (4/6)	0 (0/6)	33.3 (2/6)	16.7 (1/6)

When evaluating viral antigen in sham-vaccinated macaques, immunoreactivity was noted in capillary endothelial cells of the lung, pulmonary macrophages, rare type II pneumocytes, hepatocytes and Kupffer cells of the liver, macrophages in splenic lymphoid follicles, endocrine cells in both the adrenal cortex and medulla, and within stromal and germinal components of the gonad in the reproductive tract (Figures 5E, 5M, and S1). Of interest, despite presence of viral antigen, no striking or significant histologic lesions were noted in reproductive tissues of the sham-vaccinated group. In all cases, immunoreactivity was observed to be more widely dispersed and in larger numbers of cells than lesions observed on histopathology. In pNP-vaccinated macaques, immunoreactivity was observed in hepatocytes (Figure 5F), rare macrophages in splenic follicles (Figure 5N), and adrenal cortical endocrine cells of 50% of evaluated sections (Figure 5V). Immunoreactivity within the pGPC-vaccinated macaques was noted within Kupffer cells within the liver (Figure 5G), macrophages and follicular dendritic cells of the spleen (Figure 5O), and within endocrine cells of the adrenal cortex and medulla of 50% of the evaluated adrenal glands (Figure 5W). Immunoreactivity was not detected in any evaluated tissue in pNP + pGPC-vaccinated macaques (Figures 5H, 5P, and 5X). Presence of viral antigen in the adrenal gland in sham-, pNp-, or pGPC-vaccinated animals was not associated with pathology (Figures 5Q–5T).

## Discussion

A safe, efficacious vaccine to prevent CCHF is critically needed to address the significant morbidity and mortality caused by CCHFV infections. Although multiple vaccine platforms have been evaluated in rodent models,^9^ our DNA vaccine platform is, to date, the only vaccine with demonstrated efficacy in NHPs.^12^ In a previous study, a three-dose vaccine regimen with pNP + pGPC was immunogenic, and animals were protected from clinical signs of disease and had significantly reduced viral loads in every tissue evaluated. Our current data suggest that a dose-sparing prime-boost vaccination is sufficient to confer significant protection against CCHFV challenge. This is an important consideration, as countries where CCHFV is endemic may have limited healthcare resources for vaccination campaigns or when rapid immunity is needed to address an outbreak. A DNA vaccine delivered by a jet injector device was recently approved in India with a three-dose regimen.^15^ Pre-clinical data supported the three-dose regimen, and it was found to generate a 66% vaccine efficiency against the SARS-CoV-2 Delta variant.^15^ In animal models tested with the ZyCOV-D DNA vaccine, the third dose improved antibody levels.^16^^,^^17^ This is similar to our own observation with a COVID-19 DNA vaccine, where three doses improve immunogenicity.^17^ However, with our CCHF DNA vaccine, the antibody responses seemed to peak already after two doses.^12^ This may reflect the high intrinsic immunogenicity of the CCHF NP. Thus, the immunogenicity of different antigens may differ despite a similar delivery technology.

Our data suggest that the immune responses to both pNP and pGPC antigens participated to give the best protection against disease. Animals receiving both pNP + pGPC had earlier control of viremia, viral loads that consistently trended lower in multiple tissues, and no evidence of pathology in the liver nor presence of viral antigen in multiple tissues. Furthermore, our data suggest that pNP may provide greater protection on its own compared with pGPC, as we found that pNP-only, but not pGPC-only, vaccination could significantly reduce viral RNA burdens in two key targets of CCHFV pathology: the liver and spleen. Overall viral burdens in pGPC-only animals trended higher than pNP-only animals. However, control of viremia and reduction of viral burden in several tissues was achieved in all groups, suggesting that immunization with NP and/or GPC can confer protective immunity. These data are consistent with studies in mice showing that GPC- or NP-only vaccines can be protective.^10^^,^^13^^,^^18^^,^^19^^,^^20^

Our data from pNP-only-vaccinated animals also suggest that inclusion of the GPC in vaccine preparations is dispensable for protection against CCHFV infection in NHPs. Similarly, studies evaluating NP-only vaccine platforms have reported significant protection in lethally infected mice.^10^^,^^13^^,^^21^ However, the vaccine platform expressing the NP may be an important consideration, as a modified vaccinia virus vaccine expressing the NP was immunogenic but failed to confer protection.^22^ Nevertheless, our data indicate that vaccination with both antigens provided the most robust control of the CCHFV challenge, suggesting that both antigens contribute to optimal protection.

We can infer from our data that CCHFV-specific antibody may be the primary correlate of protection against CCHFV. First, pGPC-only-vaccinated animals had significant CCHFV-specific cellular immunity but no detectable CCHFV-specific IgG prior to CCHFV infection. This predominantly cellular immunity correlated with significantly higher viral burdens in several tissues, including the liver, compared with pNP + pGPC-vaccinated animals, which had both humoral and cellular responses against CCHFV. Second, although pNP- and pNP + pGPC-vaccinated animals had similar titers of CCHFV-specific antibody prior to CCHFV infection, pNP + pGPC-vaccinated animals had reduced signs of clinical disease, earlier control of viremia, reduced antigen by immunohistochemistry (IHC) in the liver, and significantly less viral RNA in the liver compared with pNP-only-vaccinated animals. This correlated with significant, albeit low, cellular immunity in the pNP + pGPC-, but not pNP-only-, vaccinated animals. Based on these observations, we hypothesize that antibodies directed against NP and cellular immunity against either the NP or GPC both contributed to efficient control of CCHFV infection in vaccinated animals. Studies evaluating the correlates of protection for a modified vaccinia Ankara-based vaccine expressing the CCHFV GPC suggested that both humoral and cellular immunity contributed to protection,^23^ while studies evaluating a replicating RNA vaccine demonstrated a key role for humoral immunity in protection.^21^

Our findings add to a growing body of evidence that although vaccine-mediated protection against CCHFV requires CCHFV-specific antibody, mechanisms beyond antibody-mediated neutralization of infectious virus are required. Multiple vaccine platforms for CCHFV have shown robust protection without detectable neutralizing antibodies or without including the exposed viral glycoproteins.^10^^,^^12^^,^^13^^,^^21^ Conversely, a subunit vaccine that induced high levels of neutralizing antibodies was unable to protect against CCHFV challenge.^24^ Neutralizing and non-neutralizing antibodies have shown protective effects,^25^^,^^26^ while similarly potent neutralizing antibodies failed to protect mice.^26^ Cumulatively, these results demonstrate that vaccine-induced or therapeutically administered neutralizing antibodies are neither necessary nor always sufficient for protection against CCHFV. Studies utilizing a monoclonal directed against GP38 demonstrated a requirement for complement activity to protect against CCHFV challenge,^27^ suggesting that antibody Fc-effector functions may be important. However, whether Fc-effector functions are how NP-directed antibodies control the virus is unclear, as GP38 is exposed on the cell and virion surface,^27^ while NP is not.^21^ The likely inaccessibility of NP to circulating antibody argues against mechanisms such as antibody-dependent complement activation or antibody-dependent cellular cytotoxicity. We also cannot exclude the possibility that the anamnestic humoral responses observed in all vaccinated groups, including pGPC, contributed to control of the infection, nor can we exclude the possibility of anamnestic cellular immunity, which was not measured.

### Our study has some important limitations

The cynomolgus macaque model used in this study is not uniformly lethal,^12^^,^^14^^,^^28^^,^^29^ and in the present study, clinical disease, even in the sham-vaccinated group, was mild. To date, there are no reported uniformly lethal NHP models for CCHF. However, this cynomolgus macaque model accurately recapitulates many aspects of human CCHF including varied and often mild disease outcome. The cumulative evaluation of vaccine candidates in stringent rodent models and NHP models will be important for continued vaccine development. Further, we delivered the vaccine via electroporation, which requires access to electricity and additional equipment beyond needles and syringes. Although we showed significant protection after two immunizations, simpler delivery methods may be preferred for the vaccination of rural populations with limited access to healthcare facilities. The separate plasmids expressing NP and GPC may also complicate clinical development, although our data suggest that protection can be conferred by the NP plasmid alone. Lastly, we did not formally investigate the correlates of protection within our study. Determining how vaccines protect against CCHFV and what immune responses must be monitored are important considerations not only for our vaccine but for other vaccine platforms as they move into clinical trials.

Cumulatively, we here extend our previous observations and show that a homologous prime-boost vaccination with plasmids expressing the CCHFV NP and GPC confer protection against CCHFV in an NHP model of CCHF. Vaccination was well tolerated, indicating the utility of the *in vivo* electroporation technique to deliver the vaccine. Furthermore, our studies suggest that although the pNP plasmid alone has protective effects, a combination of immune responses to both NP and GPC antigens may provide an overall better protection against CCHFV challenge. These data will support continued pre-clinical and clinical development of a vaccine candidate and will also help inform additional vaccination strategies to address the significant morbidity and mortality of CCHFV.

## Materials and methods

### Animals, biosafety, and ethics

All infectious work with CCHFV and sample inactivation was performed in a maximum containment laboratory in accordance with standard operating procedures approved by the Rocky Mountain Laboratories Institutional Biosafety Committee, Division of Intramural Research, National Institute of Allergy and Infectious Diseases, National Institutes of Health (Hamilton, MT, USA). All animal work was performed in strict accordance with the recommendations described in the Guide for the Care and Use of Laboratory Animals of the Office of Animal Welfare, National Institutes of Health, and the Animal Welfare Act of the US Department of Agriculture, in an AAALACi-accredited facility. Animals were housed in adjoining individual primate cages that enabled social interaction, under controlled conditions of humidity, temperature, and light (12 h light/12 h dark cycles). Water was available *ad libitum*. Animals were monitored at least twice daily (pre- and post-infection) and were scored for signs of disease daily by the same person blinded to the study groups using a previously established scoring sheet. A score (0–15) was assigned for each of the following: general appearance, skin and fur, nose/mouth/eyes/head, respiration, feces and urine, food intake, and locomotor activity. All scoring was performed prior to anesthesia for treatments and examinations. Animals were fed commercial monkey chow, treats, and fruit twice a day by trained personnel. Environmental enrichment consisted of manipulanda, visual enrichment, and audio enrichment. All procedures on NHPs were performed by board-certified clinical veterinarians who also provided veterinary oversight of the study. All necropsies were performed by board-certified veterinary pathologists.

### Plasmids and vaccinations

Plasmids were prepared and vaccinations delivered as described previously.^12^

### Virus

Animals were challenged with 1 × 10^5^ TCID_50_ of CCHFV strain Hoti divided between subcutaneous injections to the cranial dorsum and intravenously through the saphenous vein as previously described.^14^ Our challenge stock of CCHFV Hoti was propagated, titered, and sequenced as previously described.^14^^,^^30^

### ELISA

CCHFV-specific IgG responses in serum were quantified by an in-house ELISA as previously described.^12^

### Serum neutralization titers

SW13 cells were plated using L-15 Media (ATCC) supplemented with 10% fetal bovine serum, 4 mM L-glutamine, 50 μg/mL penicillin, and 50 μg/mL streptomycin in 96-well tissue culture plates for 80%–90% confluency at time of assay the next day. Sera was inactivated at 56°C for 30 min and serially diluted 1:2 starting with a 1:10 dilution in triplicate in L-15 media supplemented with 2% fetal bovine serum, 4 mM L-glutamine, 50 μg/mL penicillin, and 50 μg/mL streptomycin. Sera dilutions were mixed 1:1 with 120TCID50 CCHFV strain Hoti, and the sera-virus mixture was incubated at 37°C without CO_2_ for 1 h before 100 μL was applied to SW13 cells. Cells were incubated at 37°C and checked for serum toxicity after 24 h. Cytopathic effect (CPE) was read on day 5 and the highest dilution of sera to show no CPE recorded.

### IFNγ ELISpot

PBMCs were isolated from EDTA-treated whole-blood spun over a Histopaque 1077 gradient (Sigma). Red blood cells were lysed with ACK lysis buffer (Gibco) and PBMCs frozen in fetal bovine serum supplemented with 10% dimethyl sulfoxide (Hybrimax grade, Sigma) in liquid nitrogen vapor phase. Cryopreserved PBMCs were evaluated for IFNγ production in response to CCHFV peptides by commercial ELISpot in a 384-well format (Cellular Technologies). PBMCs were thawed and plated at 100,000–400,000 cells per well in CTL-Test media. 15-mer peptides overlapping by 11 amino acids derived from the CCHFV NP or GPC were synthesized (Genscript), resuspended in DMSO (Hybrimax-grade, Sigma), and pooled at 19–31 peptides per pool. Cells were stimulated with peptide pools at a final concentration of 1 μg/mL each peptide. As positive control, cells were stimulated with PMA/ionomycin (Biolegend) or DMSO vehicle alone. Cells were incubated for 24 h at 37°C in 5% CO_2_ before plates were developed according to manufacturer’s protocol. Spots were counted using an S6 Universal analyzer (CTL) and data normalized per 1 × 10^6^ cells. Upper limit of detection was set at 300 SFCs per well. Background was defined as number of spots in wells stimulated by DMSO vehicle alone, and this value was subtracted from counts measured in stimulated wells. Each measurement was performed in duplicate.

### Immunofluorescence

L929 cells were plated using EMEM Media (ATCC) supplemented with 10% fetal bovine serum, 2 mM L-glutamine, 50 μg/mL penicillin, and 50 μg/mL streptomycin in Ibidi 8-well tissue culture-treated slides (Thermo Fisher Scientific) for 80%–90% confluency at time of infection. Cells were infected at a multiplicity of infection of 1 with CCHFV Hoti virus the next day. After 24 h, media were removed, and cells were washed with DPBS and briefly fixed in 2% paraformaldehyde. Cells were incubated in NHP sera diluted 1:500 in permwash (PBS +0.05% saponin +0.1% BSA) for 50 min, followed by three washes with permwash and incubation with secondary Alexa Fluor 488 goat anti-human IgG (Invitrogen) diluted in permwash 1:2,000 for 45 min. Lastly, cells were washed and incubated with NucBlue Live ReadyProbes Reagent (Thermo Fisher Scientific) for 20 min in the dark before final wash and overnight fixation in 2.5% paraformaldehyde.

### qRT-PCR

Viral RNA in indicated tissues was quantified by qRT-PCR as previously described.^31^

### Blood chemistry and hematology

Hematology analysis was completed on a ProCyte DX (IDEXX Laboratories, Westbrook, ME, USA), and the following parameters were evaluated: red blood cells (RBCs), hemoglobin (Hb), hematocrit (HCT), mean corpuscular volume (MCV), mean corpuscular Hb (MCH), MCH concentration (MCHC), red cell distribution weight (RDW), platelets, mean platelet volume (MPV), white blood cells (WBCs), neutrophil count (abs and %), lymphocyte count (abs and %), monocyte count (abs and %), eosinophil count (abs and %), and basophil count (abs and %). Serum chemistries were completed on a VetScan VS2 Chemistry Analyzer (Abaxis, Union City, CA, USA), and the following parameters were evaluated: glucose, blood urea nitrogen (BUN), creatinine, calcium, albumin, total protein, ALT, AST, ALP, total bilirubin, globulin, sodium, potassium, chloride, and total carbon dioxide. The complete blood hematology and chemistry data are provided in Table S2.

### IHC

Tissues were fixed in 10% neutral buffered formalin ×2 changes for a minimum of 7 days. Tissues were placed in cassettes and processed with a Sakura VIP-6 Tissue Tek, on a 12 h automated schedule, using a graded series of ethanol, xylene, and ParaPlast Extra. Embedded tissues were sectioned at 5 μm and dried overnight at 42°C prior to staining. Sections were stained with hematoxylin and eosin, and specific anti-CCHFV immunoreactivity was detected using a rabbit anti-CCHFV N protein antibody (IBT Bioservices cat. no. 04-0011) at a 1:2,000 dilution. The secondary antibody was the ImPress VR anti-rabbit IgG polymer (Vector Laboratories cat. no. MP-6401). The tissues were processed for IHC using the a Discovery Ultra automated stainer (Ventana Medical Systems) utilizing the ChromoMap DAB kit (Roche Tissue Diagnostics cat. no. 760-159). Scoring was performed by a pathologist blinded to study groups.

### Statistics

Indicated statistical tests were performed using Prism 9 (GraphPad).

## Supplemental information

Document S1. Figures S1–S6 and Table S1

Table S2. Complete blood chemistry and hematology

Document S2. Article plus supplemental information
