# Supplementary material for: Accelerated DNA vaccine regimen provides protection against Crimean-Congo hemorrhagic fever virus challenge in a macaque model
Source: Mol Ther. 2022 Oct 3;31(2):387–97. doi: 10.1016/j.ymthe.2022.09.016 (PMC9931546; doi:10.1016/j.ymthe.2022.09.016)
Supplement: Document S1. Figures S1–S6 and Table S1 [file mmc1.pdf]

YMTHE, Volume *31*

Supplemental Information

Accelerated DNA vaccine regimen provides protection against Crimean-Congo hemorrhagic fever virus challenge in a macaque model

David W. Hawman, Kimberly Meade-White, Shanna Leventhal, Sofia Appelberg, Gustaf Ahlén, Negin Nikouyan, Chad Clancy, Brian Smith, Patrick Hanley, Jamie Lovaglio, Ali Mirazimi, Matti Sällberg, and Heinz Feldmann

Supplemental Table 1: Study Participation

Study	Group	Study #	Sex	Birthdate	Weight (kg)
1	Sham	81	Male	8/21/2018	1.98
		82	Female	10/9/2018	1.93
		83	Female	9/23/2018	2.22
	pNP + pGPC	84	Male	8/15/2018	2.54
		85	Male	12/14/2018	2.32
		86	Female	8/27/2018	2.27
		87	Female	8/14/2016	2.78
		88	Male	11/22/2018	2.19
		89	Female	9/30/2017	2.26
2	Sham	90	Female	9/20/2018	2.30
		91	Female	10/28/2018	2.04
		92	Female	10/3/2016	3.10
	pNP	93	Female	6/2/2017	2.39
		94	Male	3/17/2018	2.61
		95	Male	11/20/2018	2.52
		96	Male	9/8/2018	2.48
		97	Female	2/13/2018	2.35
		98	Male	12/3/2018	2.21
	pGPC	99	Female	6/25/2017	2.14
		100	Male	9/25/2017	3.12
		101	Male	7/16/2018	2.44
		102	Female	12/31/2016	2.62
		103	Male	9/13/2018	2.79
		104	Female	11/25/2018	2.13

Supplemental Table 2: Complete Blood Chemistry and Hematology

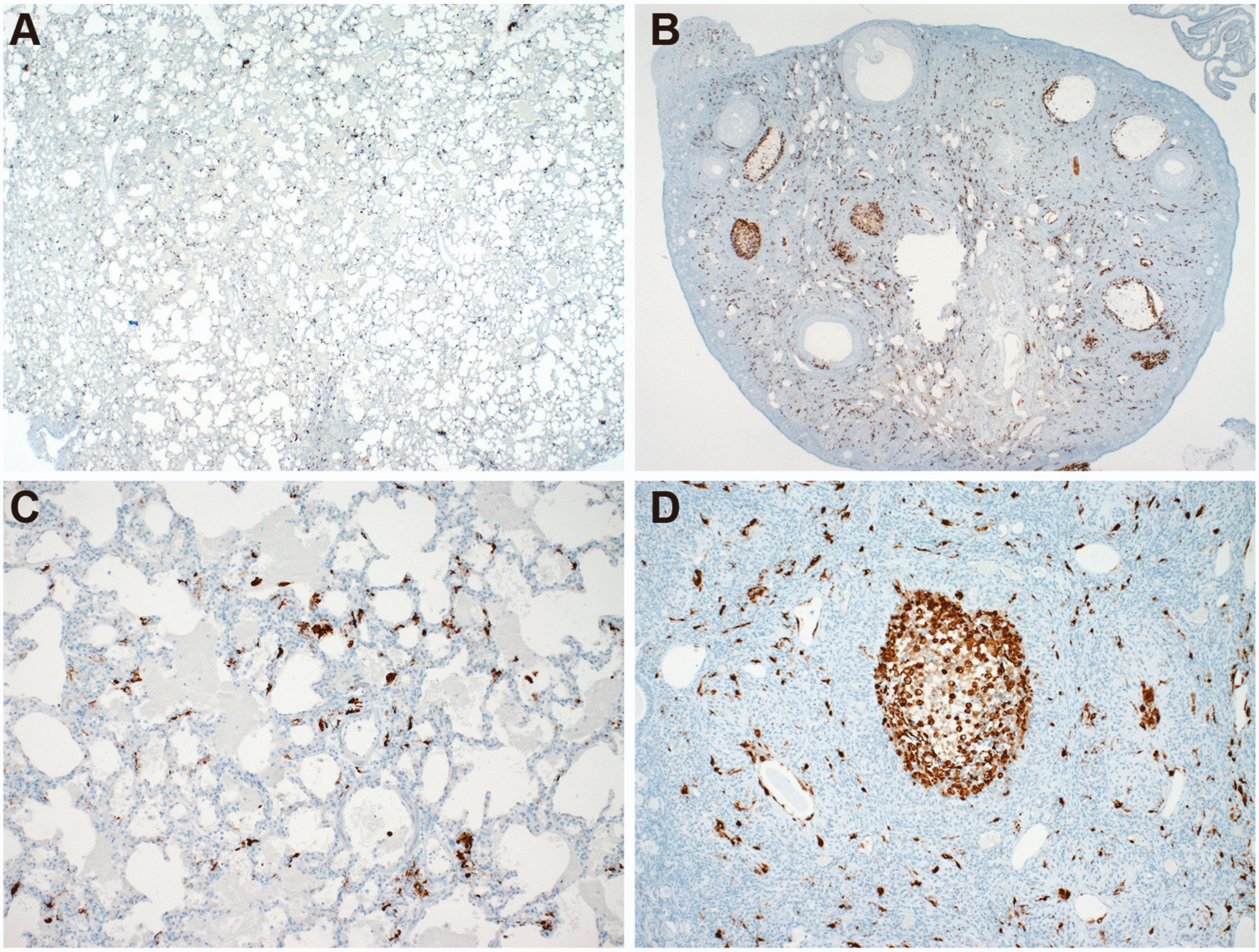

Supplemental Figure 1: Immunoreactivity in lungs and reproductive tissue. At time of necropsy, tissues from sham-vaccinated animals were formalin fixed, paraffin embedded and sectioned. Immunoreactivity was observed in the lungs (A & C) and gonads (B & D). (A - B) 100x. (C - D) 200x.
